# Induction of Primordial Germ Cells from Pluripotent Epiblast

**DOI:** 10.1100/tsw.2002.155

**Published:** 2002-03-26

**Authors:** Ying Ying, Xiaoxia Qi, Guang-Quan Zhao

**Affiliations:** Cecil H. and Ida Green Center for Reproductive Biology Sciences, Department of Pharmacology, University of Texas Southwestern Medical Center, Dallas, TX 75390-9051, USA; Division of Reproductive Endocrinology and Infertility, Women and Infants Hospital of Rhode Island, Providence, RI 02905, USA

**Keywords:** mammals, mouse, gastrulation, epiblast, germ cells, germ cell development, PGC (primordial germ cell), totipotency, pluripotency, BMP (bone morphogenetic protein), BMP2, BMP4, BMP8B, BMP receptors, SMAD1, SMAD5

## Abstract

The formation of germ cells during embryogenesis bears the ultimate importance for the continuation of every species. It becomes evident that mechanisms governing germ cell fate specification are not well conserved across the animal kingdom. In most of the invertebrate and nonmammalian vertebrate species, certain maternally derived factors are key to the establishment of germ cell lineage. In contrast, mouse primordial germ cells (PGCs) are induced from the pluripotent epiblast cells before and during gastrulation by the extraembryonic cell-derived signals. The molecular identity for some of these signals has recently been revealed by genetic and epiblast culture experiments. Both bone morphogenetic proteins 4 (Bmp4) and 8b (Bmp8b) are expressed in the extraembryonic ectoderm and are required for PGC formation. Furthermore, BMP4 or BMP8B alone are unable to induce PGCs from cultured epiblasts, while they can in combination, indicating they signal through separate receptor complexes. In addition, Bmp4 homozygous embryos cannot be induced to form PGCs by the synergistic action of BMP4 and BMP8B, suggesting that BMP4 proteins produced by pregastrula embryos are required for epiblast cells to maintain pluripotency. Moreover, Bmp2, a close relative of Bmp4, is expressed in visceral endoderm at the time of PGC specification, and inactivation of Bmp2 results in a reduction in PGC number, revealing a novel function of visceral endoderm in PGC generation in the mouse.

## INTRODUCTION

During sexual reproduction, the union of a sperm cell and a mature oocyte leads to the formation of a zygote that contains all necessary genetic materials from both parents. In nearly all species, the establishment of germ cell lineage is the most important event and usually occurs at early stages of embryonic development. A significant amount of data has been accumulated in germ cell fate specification in the invertebrate species, such as the *Drosophila* and *Caenorhabditis elegans*, through extensive genetic studies. Recent studies on PGC formation in mice with embryological and genetic approaches have greatly increased our understanding of germ cell fate determination in mammals. However, it becomes evident that mechanisms governing germ cell fate specification are not well conserved across the phylogeny. The pathways governing PGC generation in different species are significantly different, even among the vertebrate species[1,2,3,4,5,6,7,8]. In *C. elegans, Drosophila*, fish, most amphibians, and avian species, it appears that asymmetric distribution of maternally derived factors before gastrulation play crucial roles in the establishment of germ cell lineage. However, in urodeles and mammals, PGCs are induced from pluripotent stem cells during gastrulation by signals derived from the extraembryonic cells. In this review, we will focus on our contemporary understanding of the generation of PGCs in mammals, using the mouse as a model system.

## GERM CELL FORMATION IN THE MOUSE

Between 16–24 h following sperm penetration, the fertilized mouse egg begins to cleave to give rise to blastomeres. Up to 8-cell stage, all blastomeres are considered equally totipotent since each is capable of generating all cell types including trophoectoderm. However, at 16-cell stage, only cells at the center retain pluripotency to form inner cell mass (ICM) and cells at the periphery either lose pluripotency or gain potency to eventually become trophoectoderm at the blastocyst stage (32 cells at embryonic day 3.5 [E 3.5]). At E 4.5, a third cell type, primitive endoderm, appears on the surface of ICM. Primitive endoderm becomes the extraembryonic parietal and visceral endoderm while ICM becomes the epiblast[9,10]. At the beginning of gastrulation (E 6.0–6.5), epiblast is a cup-shaped tissue containing pluripotent cells that can give rise to the whole fetus as well as the extraembryonic mesoderm. On top of the epiblast is the extraembryonic ectoderm that will form the major part of the placenta. It is worth noting that ICM and epiblast cells, or their derivative embryonic stem (ES) cells, are regarded by many scientists as pluripotent since it is believed that these cells cannot give rise to trophoectoderm. PGCs are first recognized as a cluster of cells located in the extraembryonic region of the primitive streak for their expression of membrane-bound alkaline phosphatase (AP) at E 7.25–7.5[11] (Fig. 1). They subsequently migrate through the base of allantois at E 8.0–8.5, the endoderm of the hindgut at E 8.5–9.5, the mesenchyme of the mesentery at E 9.5–10.5, and finally to the genital ridge by E 10.5.

During mammalian embryonic development, totitpotency and pluripotency resides within the following cells: fertilized egg or zygote, blastomeres, ICM, and many cells within the epiblast. PGCs in culture can form embryonic germ (EG) cells that share many of the same characteristics as ICM- or epiblast-derived ES cells. These characteristics include the ability to contribute to all three germ layers in embryonic chimeras[12]. Therefore, PGCs, especially the migratory ones, are also regarded as pluripotent by many researchers. This notion is further supported by the embryonic expression of a POU domain- and homeodomain-containing transcription factor, OCT4, in all of the above totipotent and pluripotent cells, including PGCs[13,14,15,16].

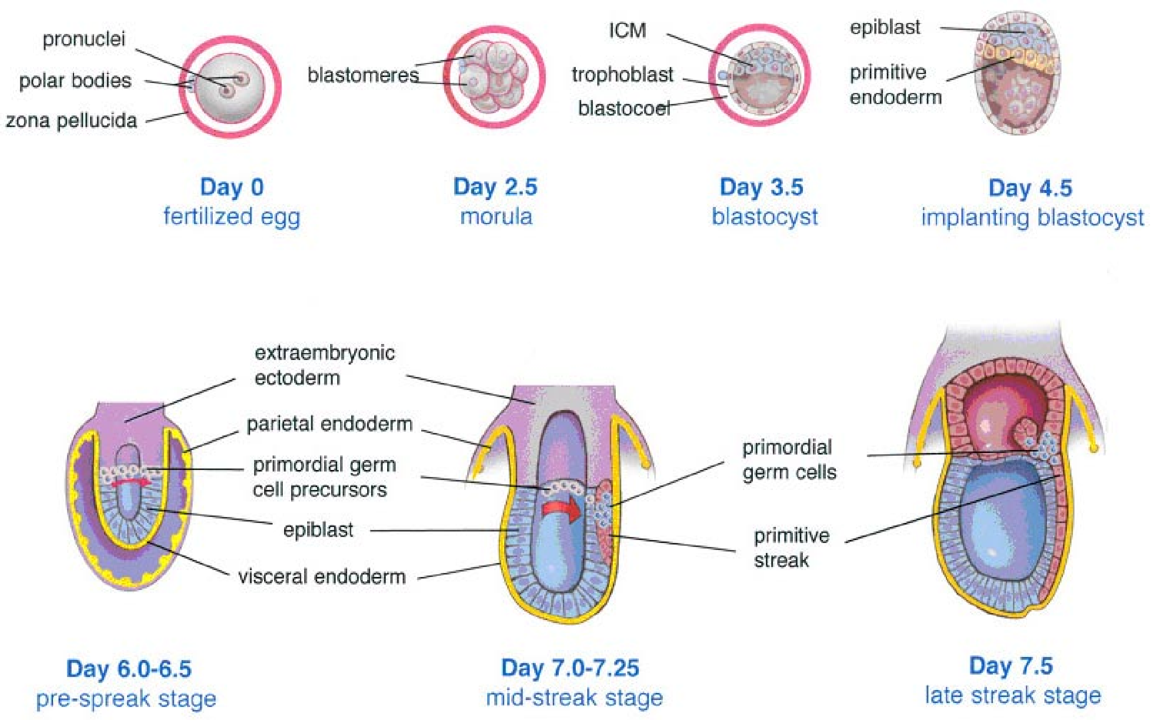

**FIGURE 1.** Establishment of germ cell lineage in the mouse. The developmental sequence of a mouse embryo from a fertilized egg (embryonic day 0) to gastrulation, the time of primordial germ cell formation at embryonic day 7.25–7.5 is shown. PGC precursors localized in the proximal epiblast and definitive PGCs in the primitive streak area are depicted as pink and blue circles with darker smaller circles as nuclei, respectively. PGC precursors migrate posteriorly into primitive streak and segregate into PGCs and mesoderm (red cells).

Since strict germ cell lineage or germ cell determinant cannot be detected in preimplantation mouse embryos, efforts have been made to further investigate PGC formation in postimplantation stages. Fate-mapping studies of the mouse embryos at the gastrula stage showed that the precursors of most tissues are derived from certain regions of the epiblast[17,18,19,20]. For example, cells from the proximal epiblast give rise to extraembryonic mesoderm while cells in the distal region of the epiblast will form the neuroectoderm. Lawson and Hage, using fate-mapping techniques, found that PGC precursors are located in the proximal epiblast within one- or two-cell diameter of the extraembryonic ectoderm before E 6.5[21]. Importantly, descendants of labeled single cells in the proximal epiblast were found in PGCs and in several other lineages including allantois, yolk sac, and amnion. Therefore, the fate of germ cells is not fixed before gastrulation. Based on the above findings, Tam and Zhou carried out a series of epiblast transplantation experiments in the mouse embryos, including homotypical transplantation (proximal-to-proximal and distal-to-distal epiblasts) and heterotypical transplantation (proximal-to-distal and distal-to-proximal epiblasts)[22]. Their results showed that before E 6.5, cells of the distal epiblast (which normally give rise to the neuroectoderm and surface ectoderm) are able to generate PGCs if they are positioned in close proximity with the extraembryonic ectoderm. However, the proximal epiblast cells never form PGCs if they are transplanted into the distal region (far away from the extraembryonic ectoderm). Therefore, before E 6.5, epiblast cells at different locations have the potency to generate PGCs only if they are in close proximity to the extraembryonic ectoderm. These results indicate that the specific local signals emitted by the extraembryonic ectoderm are critical for the induction of PGCs or PGC precursors. This postulate is further supported by the coculture of epiblasts with extraembryonic ectoderm[23]. PGCs are formed within the cultured distal epiblast cells only in the presence of the extraembryonic ectoderm, but not if epiblasts are cultured with STO feeder cells alone.

## PGC-INDUCING SIGNALS

The above studies provide solid embryological evidence that PGC formation in the mouse (possibly most mammals) is induced by signals from the extraembryonic ectoderm rather than predetermined by maternal factors. Therefore, the further identification and characterization of such signals should advance our understanding of the mechanisms governing germ cell formation and even the maintenance of totipotency or pluripotency. As shown by Lawson et al. and Ying et al., genes encoding BMP4 and BMP8B, two growth factors of the BMP family, are similarly expressed in the extraembryonic ectoderm at E 5.5–7.5[24,25]. Very importantly, embryos with targeted deletion of *Bmp4* never form PGCs, while *Bmp8b* null mutants have no or very few PGCs. Thus, these two genes are expressed in the right place and time to serve as the extraembryonic ectoderm-derived signals for PGC precursor formation.

BMPs are members of the transforming growth factor β (TGFβ) superfamily[26,27]. Based on their sequence identity at amino acid level, these proteins are grouped into several classes. The 60A and DPP classes are among the best characterized. The 60A class includes the *Drosophila* 60A protein and the vertebrate BMP5, BMP6, BMP7, BMP8A, and BMP8B proteins, while the DPP class consists of the *Drosophila* DPP and the vertebrate BMP2 and BMP4. BMPs are synthesized as precursors with a large N-terminal prodomain and a C-terminal mature domain. The monomers of BMP proproteins dimerize intracellularly through disulfide bonds and the mature region is released as a result of the proteolytic cleavage during secretion. Therefore, BMPs (like other TGFβ superfamily members) function as homodimers or heterodimers to elicit their biological activities in cell proliferation, cell survival, differentiation, and cell fate determination[26]. For example, in *Drosophila*, the maternal DPP is required for dorsal-ventral patterning at blastoderm stage, while the zygotic expression is required for the development of the heart, midgut, eye, and appendages[28]. Mice deficient in BMP2 or BMP4 die before mid-gestation due to defects in mesoderm formation and heart development[29,30]. Several members of the 60A class also play important roles during embryonic development and organogenesis. For example, targeted inactivation of *Bmp7* results in defects in the kidney due to the failed proliferation, survival, and/or differentiation of the nephrogenic mesenchyme, as well as defects in eye and bone formation[31,32].

BMPs trigger cellular responses by binding to heteromeric receptor complexes that contain type I and type II receptor subunits. Both receptor types are serine/threonine protein kinases and both are essential for signal transduction[33,34,35,36]. Type II receptors have constitutively active kinase activity that phosphorylate type I receptors upon ligand and receptor complex formation. The phosphorylated type I receptors relay signals to downstream target proteins. Several putative receptors have been identified for their ability to transduce BMP signals, including type I receptors ALK1, ALK2, ALK3 (BMPR and BMPRIA), and ALK6 (BMPRIB), and type II receptors BMPRII (TALK), ActRIIA, and ActRIIB[37,38,39,40,41,42,43,44,45,46].

Among the type I receptors, *Alk3* is the only one clearly shown to be expressed in the epiblast. *Alk6* is not expressed until E 9.5 in the mouse embryo, and its mutants do not show obvious germ cell defects, suggesting that it is unlikely for *Alk6* to play a role in PGC formation[37,47]. *Alk2* is mainly expressed and functions in visceral endoderm during gastrulation[48,49]. The expression of *Alk1* during gastrulation has not been reported; inactivation of *Alk1* resulted in a phenotype similar to that of Tgfβ1 mutants in angiogenesis, suggesting that ALK1 may serve as the receptor for TGFβ family[50]. Thus, ALK3 seems to be the best candidate of type I BMP receptor in PGC induction since it is expressed in the epiblast and mutation in *Alk3* causes embryonic lethality before mesoderm or PGC formation[46]. All three putative type II receptors for BMPs (*BmprII*, *ActrIIa*, and *ActrIIb*) are expressed in the epiblast during gastrulation[51,52]. Mutation in *BmprII* results in a phenotype very similar to that of *Alk3* mutants[51]. Therefore, the possible functions of *Alk3*, *BmprII*, *ActrIIa*, and *ActrIIb* in PGC formation deserve further investigation.

SMAD proteins have been shown to play pivotal roles in the signal transduction of the TGFβ superfamily[27,41]. It has also been shown that different SMADs transduce signals for different subfamilies of this superfamily. For example, SMAD1, 5, and 8 transduce signals of BMPs, while SMAD2 and SMAD3 transduce signals of TGFβs and activins. *Smad1* and *Smad5* are expressed in the proximal epiblast cells during gastrulation while *Smad8* is mainly expressed in allantois[53,54,55]. Thus, it is likely that SMAD1 and SMAD5 serve as the signal transducers for BMP4 and BMP8B during PGC induction. This notion is supported by the recent findings that targeted mutations in either of them resulted in the absence of PGCs or a severe reduction PGC number[54,55].

## Two BMP Pathways are Required and Sufficient for PGC Induction from Cultured Epiblasts

*Bmp4* and *Bmp8b* are expressed in the extraembryonic ectoderm, and both are required for PGC generation in the mouse. In order to investigate why two similar genes are required for the same PGC induction process, Ying et al.[25] first examined whether one gene is required for the expression of the other. Their *in situ* hybridization experiments did not reveal a significant change of Bmp8b expression in Bmp4 homozygous mutant embryos at E 6.5 in comparison with that of the wild-type embyos, and the same was true for *Bmp4* expression among *Bmp8b* mutants. Thus, it is unlikely that one is required for the expression of the other[25].

*Bmp4* heterozygotes show a 50% reduction in PGC number compared with wild-type embryos on several genetic backgrounds as a result of the haploid insufficiency[24]. Any further compromise in BMP4 signaling pathway in these heterozygotes, including reduction in ligands, receptors, or SMADs, should exacerbate the phenotype of PGC defects in these mutants. Therefore, if BMP4 and BMP8B function similarly or equally through the same receptor complexes, mutation in *Bmp8b* should exacerbate the mutant phenotype of *Bmp4* heterozygotes. As shown by Ying et al., there is no obvious difference in PGC numbers between Bmp4+/- mutants and *Bmp4*+/-; *Bmp8b*+/- double mutants[25]. These results show that BMP4 and BMP8B proteins do not have an additive effect in PGC induction and further point to two possible scenarios. First, BMP4 and BMP8B function as homodimers through separate receptor complexes. Second, it has been shown that the heterodimers of DPP and 60A classes are much more potent in mesoderm and bone induction[56,57,58]. Thus, BMP4/BMP8B heterodimers may be the true functional ligands in the process of PGC induction.

To distinguish between the above possibilities, Ying et al. established an *in vitro* system to coculture the epiblasts of E 6.0–6.25 mouse embryos with COS cells producing BMP4 and/or BMP8B proteins[59]. Their results showed that PGCs can be induced from the cultured epiblasts by the COS cells producing both BMP4 and BMP8B, but not by COS cells producing BMP4 or BMP8B alone. Furthermore, similar PGC induction results were obtained with COS cells expressing both BMPs (homodimers and possible heterodimers produced) and with a mixture of equal numbers of BMP4-producing COS cells and BMP8B-producing COS cells (only homodimers produced). These data clearly indicate that BMP4 and BMP8B homodimers are not only required, but their combination is sufficient to induce PGCs from the cultured epiblasts. It is also indicated that these two proteins must signal through separate receptor complexes. Moreover, BMP4 and BMP8B homodimers act directly on the epiblast cells since PGCs can be induced from the isolated epiblast masses in the absence of the visceral endoderm.

## Two Sources of Inductive Signals

Embryological studies with lineage tracing and epiblast transplantation support a model of PGC induction from the proximal epiblast cells by the extraembryonic ectoderm-derived signals[21,22]. Results from *in vivo* genetic studies and *in vitro* epiblast cultures have validated this model and have further revealed that BMP4 and BMP8B are not only required but also sufficient for PGC induction in the mouse[24,25,59]. During and before gastrulation, in addition to the extraembryonic ectoderm, the visceral endoderm is in close contact with epiblast as well. Knowing that visceral endoderm expresses BMP2[60], a very close relative of BMP4, Ying and Zhao tested whether BMP2 participates in PGC induction[62]. Their data revealed that *Bmp2* is expressed at detectable levels in the visceral endoderm after E 6.0, especially around the boundary region between the epiblast and the extraembryonic ectoderm. The levels of *Bmp2* expression appear to increase as the embryo develops from E 6.0 to E 7.0. Furthermore, a gradient is formed with the highest levels at the posterior and the lowest levels at the anterior. Significantly, *Bmp2* homozygous mutants show a 50% reduction in PGC number attributable to defects in PGC formation. Therefore, a role of visceral endoderm, as a second source of BMPs, in PGC formation has been established during mouse embryogenesis. These genetic studies have also revealed that mutation in *Bmp2* exacerbates the PGC defects of *Bmp4* heterozygotes; thus, these two genes have an additive effect in PGC induction. However, neither *Bmp2* nor *Bmp4* has an additive effect with *Bmp8b*. These data suggest that BMP2 and BMP4 signal directly through the same or similar receptors on the epiblast that are different from those of BMP8B. The biological significance of the anterior-posterior gradient of *Bmp2* expression in visceral endoderm is not yet clear. It is possible that such a gradient helps to maintain sufficient levels of BMP2/4 along the migratory path of PGC precursors. Alternatively, BMP2/4 may serve as chemotactic factors to pluripotent stem cells or PGCs precursors; thus, a gradient of BMP2 may guide PGC precursors to the primitive streak. The latter hypothesis is supported by the fact that PGCs are largely trapped in the yolk sac if Bmp4 is not expressed in the extraembryonic mesoderm[61].

## Two Steps of Induction

The combination of genetic studies and *in vitro* epiblast cultures strongly supports a notion that the DPP class of BMPs and the 60A-member BMP8B signal as homodimers through separate receptor complexes in PGC precursor induction. Therefore, it is anticipated that defects in PGC formation in *Bmp8b* and *Bmp4* null mutants can be rescued by BMP8B and BMP4 proteins. As shown by Ying et al., COS cells expressing BMP8B proteins indeed rescue the PGC defects of *Bmp8b* mutants as revealed by AP staining[59]. Surprisingly, similar experiments with COS cells producing BMP4 proteins fail to induce PGCs from *Bmp4* null mutants. Moreover, *Bmp4* null embryos (E 6.0–6.25) fail to produce any PGCs even in the presence of COS cells producing both BMP4 and BMP8B, although the same COS cells can induce PGCs from isolated wild-type epiblast masses[59]. These results clearly reveal that BMP4 proteins are required prior to E 6.0 for epiblast cells to gain or maintain germline competency before the synergistic action of BMP4 and BMP8B. Bmp4 expression is detected in the inner cell mass at E 3.5, and is then gradually switched to the extraembryonic ectoderm by E 5.5[24,60]. Thus, the epiblast cells and their precursors have already been or are continuously exposed to BMP4 prior to E 6.0. These data indicate that at least two steps of BMP signaling are required to induce PGCs or to maintain the status of pluripotency: (1) BMP4 is required before E 6.0 and (2) BMP2/4 and BMP8B are required after E 6.0 for the establishment of germ cell lineage in the mouse (Fig. 2).

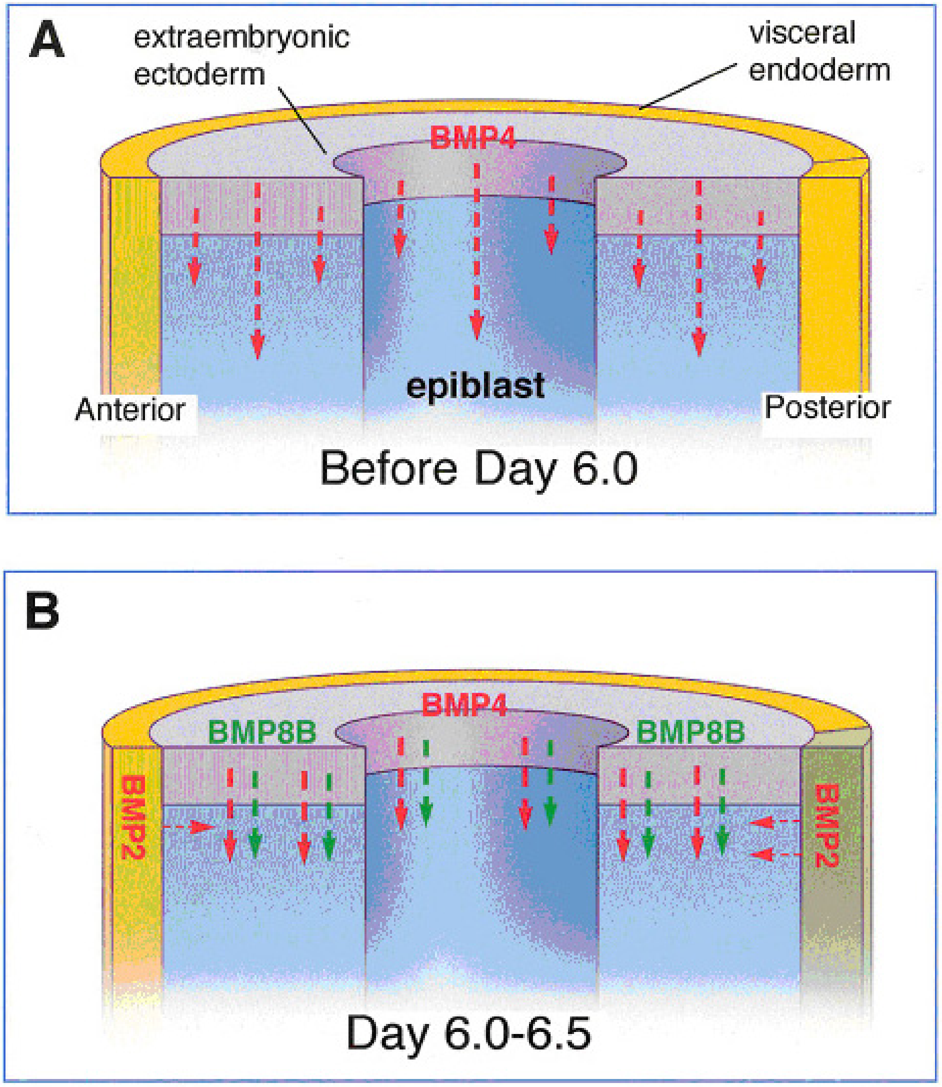

**FIGURE 2.** Two steps of PGC induction by multiple BMPs during mouse embryogenesis. The diagrams only show one half of the mid portion of a pregastrula embryo. (A) Before E 6.0, BMP4 produced by the extraembryonic ectoderm is required for the epiblast cells to maintain pluripotency or germ cell competency that is essential for subsequent inductions. (B) At E 6.0–6.5, BMP4 and BMP8B homodimers directly bind to separate receptor complexes on cells within the proximal epiblast to synergistically instruct them to develop toward a germ cell fate. In addition, visceral endoderm-derived BMP2 acts in a similar way as BMP4 for germ cell induction. Visceral endoderm at the posterior region expresses higher levels of BMP2 (two small red arrows) than the anterior region (one small red arrow). BMP2 and BMP4 are indicated in red, while BMP8B is depicted in green.

## CLOSING REMARKS

The temporal and spatial expression patterns of *Bmp2*, *Bmp4*, *Bmp8b*, *Smad1*, *Smad5*, and their respective mutant phenotypes have established that two BMP signaling pathways (BMP2/4 pathway and BMP8B pathway) are required for PGC formation in the mouse. These two pathways are also sufficient to induce PGCs from cultured epiblasts. Nevertheless, the molecular links (receptor complexes) between BMPs and SMAD proteins remain elusive. It is not clear what receptor subunits participate in BMP2/4 or BMP8B signal pathways and whether these two pathways share common subunits. It also remains to be determined whether BMP2/4 and BMP8B signal through distinct SMAD proteins or converge on both SMAD1 and SMAD5. Furthermore, it is not clear what the target genes are downstream of SMAD1 and SMAD5 in the proximal epiblast cells. Finally, since Oct4 is functionally required for pluripotency and consequently for PGC formation, the relationship of BMP signaling and OCT4 activity should also be an important question. It appears that such possible connection between BMPs and OCT4 is further complicated by germ cell nuclear factor (GCNF) that directly suppresses the Oct4 expression[63]. All of these questions will be addressed in the future.

## ACKNOWLEDGEMENTS

This work is supported by grants from the National Institute of Child Health (HD-36218 and HD-39154) and the Basil O'Connor Starter Scholar Research Award to G.-Q. Zhao.
